# Chemical interaction mechanism of 10-MDP with zirconia

**DOI:** 10.1038/srep45563

**Published:** 2017-03-30

**Authors:** Noriyuki Nagaoka, Kumiko Yoshihara, Victor Pinheiro Feitosa, Yoshiyuki Tamada, Masao Irie, Yasuhiro Yoshida, Bart Van Meerbeek, Satoshi Hayakawa

**Affiliations:** 1 Advanced Research Center for Oral and Craniofacial Sciences, Okayama University Graduate School of Medicine, Dentistry and Pharmaceutical Sciences, 2-5-1 Shikata-cho, Kita-ku, Okayama 700-8558, Japan; 2 Center for Innovative Clinical Medicine, Okayama University Hospital, 2-5-1 Shikata-cho, Kikta-ku, Okayama 700-8558, Japan; 3 Department of Restorative Dentistry, School of Dentistry, Federal University of Ceará, Fortaleza, Brazil; 4 Department of Occlusal and Oral Functional Rehabilitation, Okayama University Graduate School of Medicine, Dentistry and Pharmaceutical Sciences, 2-5-1 Shikata-cho, Kita-ku, Okayama 700-8558, Japan; 5 Department of Biomaterials, Okayama University Graduate School of Medicine, Dentistry and Pharmaceutical Sciences, 2-5-1 Shikata-cho, Kikta-ku, Okayama 700-8558, Japan; 6 Department of Biomedical, Dental Materials and Engineering, Graduate School of Dental Medicine, Hokkaido University, Kita 13, Nishi 7, Kita-ku, Sapporo 060-8586, Japan; 7 KU Leuven (University of Leuven), Department of Oral Health Sciences, BIOMAT & University, Hokkaido Hospitals Leuven, Dentistry, Kapucijnenvoer 7, block A–box 7001, BE-3000 Leuven, Belgium; 8 Biomaterials Laboratory, Graduate School of Natural Science and Technology, Okayama University, 3-1-1, Tsushimanaka, Kita-ku, Okayama 700-8530, Japan

## Abstract

Currently, the functional monomer 10-methacryloyloxy-decyl-dihydrogen-phosphate (10-MDP) was documented to chemically bond to zirconia ceramics. However, little research has been conducted to unravel the underlying mechanisms. This study aimed to assess the chemical interaction and to demonstrate the mechanisms of coordination between 10-MDP and zirconium oxide using ^1^H and ^31^P magic angle spinning (MAS) nuclear magnetic resonance (NMR) and two dimensional (2D) ^1^H → ^31^P heteronuclear correlation (HETCOR) NMR. In addition, shear bond-strength (SBS) tests were conducted to determine the effect of 10-MDP concentration on the bonding effectiveness to zirconia. These SBS tests revealed a 10-MDP concentration-dependent SBS with a minimum of 1-ppb 10-MDP needed. ^31^P-NMR revealed that one P-OH non-deprotonated of the PO_3_H_2_ group from 10-MDP chemically bonded strongly to zirconia. ^1^H-^31^P HETCOR NMR indicated that the 10-MDP monomer can be adsorbed onto the zirconia particles by hydrogen bonding between the P=O and Zr-OH groups or via ionic interactions between partially positive Zr and deprotonated 10-MDP (P-O^−^). The combination of ^1^H NMR and 2D ^1^H-^31^P HETCOR NMR enabled to describe the different chemical states of the 10-MDP bonds with zirconia; they not only revealed ionic but also hydrogen bonding between 10-MDP and zirconia.

Zirconia ceramics possess several advantageous properties such as their pleasing esthetic color, high fracture toughness, and optimal biocompatibility. Recently, yttria-stabilized tetragonal zirconia polycrystal (Y-TZP) ceramics are widely used for fixed dental prostheses and dental implants. Reliable bonding and perfect marginal integrity along the tooth-cement-zirconia interface are essential for the clinical durability of all-ceramic zirconia restorations^1^. However, it is difficult to establish a durable physico-chemical bond to Y-TZP ceramics owing to their inert surfaces and the difficulty in creating micro-retention at the surface^2^. Conventional silica-based ceramics, such as feldspatic porcelain, are best etched with hydrofluoric acid (HF) for bonding purposes. However, HF etching is less effective at zirconia^3^. Recently, several studies revealed that to be effective HF etching of zirconia requires a longer application time, a higher concentration of HF and/or a higher temperature, such as 9.5% HF at 80 °C for 1 min or at 25 °C for 1 h, or 48% HF at 25 °C for 30 min^4^, 20% or 30% HF for 30 min^5^, or 48% HF at 100 °C for 25 min^6^. Therefore, recent investigations have recommended alumina air-abrasion to increase the bond strength of composite cement to zirconia; it increases the surface area for micro-mechanical interlocking^7^,^8^. Generally, air abrasion improves (immediate) bond strength, but bond strength of 10-MDP-free cements (including cements that require beforehand application of a 10-MDP-free primer/adhesive) applied upon air abrasion was documented to decrease after thermo-cycling, while not that of a 10-MDP-containing cement (including cements that require beforehand application of a 10-MDP-containing primer/adhesive)^9^,^10^,^11^. These results indicate that a solely physical surface treatment, such as air abrasion, does not suffice to obtain a durable resin-zirconia bond.

To improve bond strength and durability of resin-based materials to zirconia, many surface treatments, such as silica tribochemical coating and silica organoprecursor deposition, were developed to facilitate chemical bonding, particularly by means of silane chemical interaction with the deposited silica^8^,^12^. In addition, phosphoric-acid ester monomers were shown to chemically bond with pure zirconia. In particular, primers and composite cements that contain 10-methacryloyloxy-decyl-dihydrogen-phosphate (10-MDP) resulted in a relatively high bond strength and durability^13^,^14^,^15^,^16^. Kitayama *et al*.^17^ and Koizumi *et al*.^18^, who evaluated the bond strength of several 10-MDP containing cements and primers to zirconia, assumed that the high bond strength to zirconia would rely on a chemical reaction of 10-MDP with zirconium oxide^17^,^18^. Although many investigations mentioned the bonding efficacy of 10-MDP to zirconia^16^ and some tried to present theoretical schemes detailing the interactions involved, very little evidence of such chemical interactions have today been demonstrated. Several studies focused on the chemical analysis of 10-MDP treated zirconia^19^. Chen *et al*.^20^ investigated chemical bonding by contact-angle measurements and time-of-flight secondary ion mass spectrometry (TOF-SIMS), whereas Kim *et al*.^21^, Pilo *et al*.^22^ and Qian *et al*.^23^ assessed the chemical bonding by Fourier transform infrared spectroscopy (FTIR). TOF-SIMS and FTIR measurements can only detect the chemical functionality of 10-MDP on the zirconia surface; however, they cannot provide direct evidence of the proposed type of chemical bonding. Xie *et al*.^24^ disclosed the chemical bond between 10-MDP and zirconia using XPS.

There exists a definitive need to clearly understand the underlying mechanisms of chemical interaction on an atomic level. The primary chemical interaction of 10-MDP with zirconium oxide must be fully elucidated, so to design acidic functional monomers with improved bonding performance in light of the ultimate aim to prolong the clinical lifetime of zirconia restorations. The aim of this study was therefore to determine the type of chemical interactions that occur between 10-MDP and zirconium oxide using ^1^H and ^31^P magic angle spinning (MAS) nuclear magnetic resonance (NMR) and two-dimensional (2D) ^1^H → ^31^P heteronuclear correlation (HETCOR) NMR. Shear bond-strength (SBS) tests were conducted to determine the effect of 10-MDP concentration on the bonding effectiveness to zirconia. The hypothesis tested was that the chemical interaction of 10-MDP with zirconium oxide relies solely on ionic interaction of 10-MDP^−^ with partially positive zirconium.

## Results

### Shear bond strength (SBS) test

**Figure 1:**
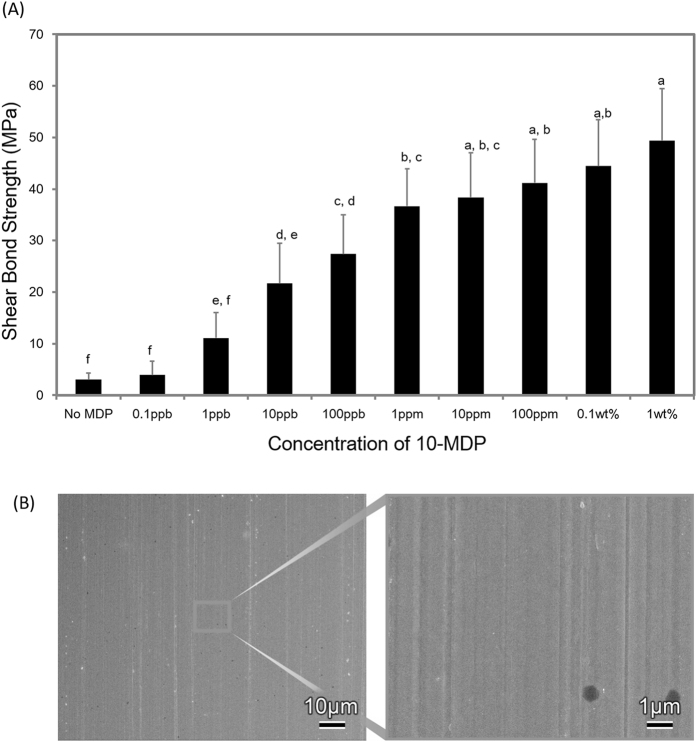
(**A**) Shear bond strength to zirconia for different concentrations of 10-MDP (mean in MPa with standard deviation). (**B**) Feg-SEM photomicrograph of the zirconia surface polished with 15-μm diamond particles at low (a) and high magnification (b).

Figure 1 presents the results of the SBS tests with different concentrations of 10-MDP applied on zirconia. The statistical analysis showed no difference between the bond strength of the control sample (without prior 10-MDP treatment) and the 0.1-ppb and 1-ppb 10-MDP specimens. However, higher concentrations of 10-MDP yielded significantly higher bond strengths with a clear concentration dependency. Fracture analysis revealed 100% ‘adhesive’ failure for the 10-MDP primers with a 10-MDP concentration of 1 ppm or below. Only half of the specimens (50%) failed ‘adhesively’ for the 10-MDP primers with a 10-MDP concentration of 10 ppm or above, with the other half (50%) having failed in a ‘mixed’ failure mode.

### NMR measurement

**Figure 2:**
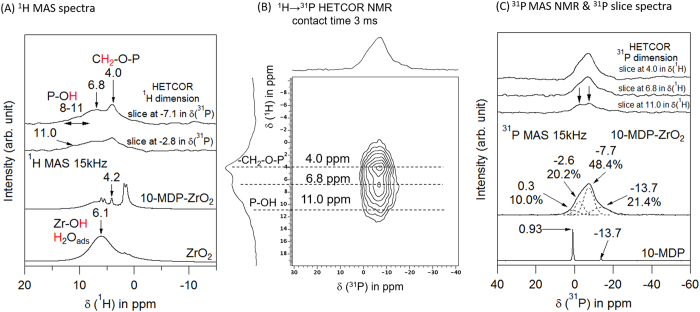
(**A**) ^1^H MAS NMR spectra of zirconium powder reacted with 10-MDP together with two characteristic slice spectra taken at −7.1 and −2.8 ppm in the ^1^H dimension of the 2D ^1^H → ^31^P HETCOR NMR spectrum obtained with a CP contact time of 3 ms. (**B**) 2D ^1^H → ^31^P HETCOR NMR spectrum obtained with a CP contact time of 3 ms. (**C**) ^31^P MAS-DD NMR spectrum of the interaction between 10-MDP and zirconia together with three characteristic slice spectra taken at 4.0, 6.8, and 11.0 ppm in the ^31^P dimension of the 2D ^1^H → ^31^P HETCOR NMR spectrum obtained with a CP contact time of 3 ms.

Figure 2A shows the ^1^H MAS NMR spectra of ZrO_2_ and 10-MDP-coated zirconia (10-MDP_ZrO_2_). A broad resonance at 5.0 ppm was assigned to the Zr-OH groups and adsorbed water molecules from the surface hydrated layer around the zirconia particles^25^. Six sharp resonances at 1.4, 1.6, 1.9, 4.1, 5.5, and 6.0 ppm and a weak broad resonance around 6 ppm were observed for the 10-MDP_ZrO_2_ specimens. The former sharp resonances were assigned to the methacryloyloxy groups of the 10-MDP monomer^26^. The latter broad resonance peak is related to various OH-groups, including not only groups at the surface hydrated layer of zirconia, but also the P-OH groups of 10-MDP.

In the ^1^H → ^31^P HETCOR NMR measurements of 10-MDP_ZrO_2_ (Fig. 2B), slices taken at −2.8 and −7.1 ppm in the ^31^P dimension showed characteristic ^1^H resonances assigned to P-OH bonds at 8–11 ppm^27^, Zr-OH bonds at 6.8 ppm^27^, and the CH_2_-O-P bonds of the methacryloyloxy groups at 4.0 ppm in δ(^1^H)^26^. These results indicate that the 10-MDP monomer can be adsorbed onto the zirconia particles via hydrogen bonding or ionic interaction between the P-OH and Zr-OH groups or between P-O^−^ and partially positive Zr, respectively.

Figure 2B shows the ^1^H → ^31^P HETCOR NMR spectrum of 10-MDP_ZrO_2_. Slices taken at 4.0, 6.8, and 11.0 ppm in the ^1^H dimension show a broad characteristic ^31^P resonance that should be assigned to the P atoms of the adsorbed 10-MDP. For comparison, the ^31^P NMR signal of the 10-MDP monomer in the acetone solution is also shown (Fig. 2C). The sharp peaks at 0.93 and −13.7 ppm in δ(^31^P) are associated with the monomer and dimer of 10-MDP, respectively^28^. The broad resonance of the ^31^P feature can be deconvoluted into four peaks around −13.7, −7.7, −2.6, and 0.3 ppm in δ(^31^P). These peaks are derived from the 10-MDP dimer and 10-MDP monomer. The weak peak at 0.3 ppm (10.0%) can be assigned to physisorbed and hydrogen-bonded 10-MDP monomers that do not cause deprotonation of any P–OH groups, while the weak peak at −13.7 ppm (21.4%) was assigned to physisorbed 10-MDP dimers that did not cause deprotonation of any P–OH groups^28^. Conversely, the strong peaks at −7.7 and −2.6 ppm were assigned to the chemisorbed 10-MDP monomer; the fraction of the chemisorbed 10-MDP monomer was 48.4% and 20.2%, respectively. The significant difference found in the ^31^P chemical shift implies that the 10-MDP monomer can interact with the surface hydrated layer of the zirconia particles, not only via hydrogen bonding but also via ionic interactions between P–O^−^ anions and positive Zr^4+^ groups.

## Discussion

This study investigated the mechanisms of chemical interaction between 10-MDP and zirconia in order to establish a surface-binding model of 10-MDP onto zirconia. The SBS data showed that higher concentrations of 10-MDP promote bond strength (Fig. 2). This finding agrees with results in the literature, demonstrating that 10-MDP affects the bonding effectiveness of a conventional composite cement to zirconia ceramics^16^. Additionally, Yoshida *et al*.^13^ reported significantly higher SBSs before and after thermo-cycling for 10-MDP treated zirconia. However, unlike our own investigation, their results did not show any significant differences for different 10-MDP concentrations. A possible explanation for this discrepancy could be the small range (0.2–0.5 wt%) of 10-MDP concentrations tested by Yoshida *et al*.^13^. Generally, commercial 10-MDP containing primers possess more than 1 wt% 10-MDP, whilst 10-MDP containing composite cements also contain higher concentrations of 10-MDP. The fact that commercial 10-MDP containing primers and cements generally achieve higher bond strengths to dental zirconia, confirms the bond-promoting effect of 10-MDP^16^,^29^.

We performed NMR analyses to gain a better understanding of the chemical state at the interface of 10-MDP with zirconia. ^1^H MAS NMR spectra of 10-MDP showed a predominant peak at 4.2 ppm. A slice taken at −2.8 ppm in the ^1^H dimension was related to P-OH at 11.0 ppm and CH_2_-O-P at 4.0 ppm. A slice taken at −7.1 ppm in the ^1^H dimension demonstrated detection of P-OH at 8–11 ppm, Zr-OH at 6.8 ppm, and CH_2_-O-P at 4.0 ppm. These findings indeed confirm the presence of phosphate groups originating from 10-MDP at the zirconia surface, even after washing with acetone, thereby indicating that the strong interaction of 10-MDP with zirconia resisted washing. Hydroxyl groups were found in the phosphate groups and at the zirconia surface; these hydroxyl groups interacted with each other. This is in agreement with results presented in a previous study^20^. When the 10-MDP containing primer Z-Prime Plus (Bisco, IL, USA) was applied onto a zirconia surface, the zirconia-surface hydrophobicity increased, as was determined by contact-angle measurements^20^. This finding is indicative of the presence of the hydrophobic 10-carbon spacer chain of 10-MDP, which coated the zirconia surface upon primer application. Furthermore, time-of-flight secondary ion mass spectrometry (TOF-SIMS) was employed in that study and disclosed the P-O-Zr chemical state upon application of Z-Prime Plus (Bisco) onto zirconia^20^.

However, the bonding state of 10-MDP to zirconia is not yet fully elucidated. Primary chemical bonding states include covalent, ionic, metallic, and chelation bonding^2^. Several bonding models between the phosphate functional group of 10-MDP and zirconia have been proposed^13^,^19^,^30^. Ultrastructural and chemical interfacial characterization have disclosed a porous phosphate-modified zirconia substrate^27^,^30^; based on ^1^H fast MAS NMR studies, the hydrogen bonding was found to have self-assembled monolayers. ^1^H and ^31^P MAS NMR were also useful to reveal the nature of the interaction of phosphoric acid with zirconia^27^,^31^; NMR provided more structural information than FTIR or Raman spectroscopy^27^. Zirconium oxide has a very heterogeneous surface with two types of surface hydroxyls that may be protonated or deprotonated as well as coordinated with the zirconium (IV) ion site, which is a very strong Lewis acid site^31^. Octadecylphosphoric acid has been reported to bind strongly to zirconia via covalent bonding^32^. 2D ^1^H → ^31^P heteronuclear correlation (HETCOR) confirmed the spectral assignment, identifying heteronuclear through-space proximities with sufficient sensitivity for ^31^P detection at all the solid specimen surfaces. The ^1^H projection of the 2D ^1^H → ^31^P HETCOR NMR spectrum showed a small shoulder at 11.0 ppm due to the P-OH proton (Fig. 2B), which is engaged in hydrogen bonding with either the zirconium oxide surface or with neighboring phosphate groups^27^.

In addition, slice spectra taken at 6.8 ppm due to Zr-OH and at 11.0 ppm due to P-OH in the ^1^H dimension show at least two peaks at −2.6 and −7.7 ppm that can be assigned to different chemical bonding arrangements (Fig. 2C). This indicates that in the adsorbed 10-MDP monomer the P-OH group is in close proximity of the Zr-OH groups at the zirconium oxide surface.

A weak P-OH proton signal was still observed after washing with acetone. This result is in agreement with previously published results^27^. Such hydrogen-bonding interactions between neighboring adsorbed phosphoric-acid groups from 10-MDP was reported for the first time in the present investigation and was previously only found for other acids^27^. The small shoulder peak at −13.7 ppm was assigned to the 10-MDP dimer^28^. This dimer may only be physisorbed onto zirconia.

**Figure 3:**
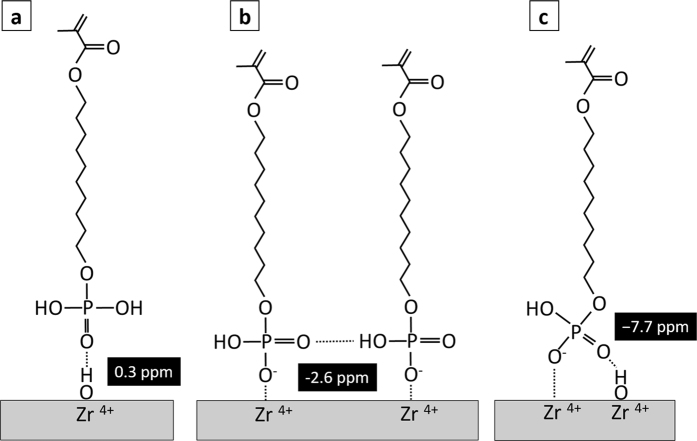
Schematic explaining the interactions of 10-MDP with zirconium and with the hydrated layer at the zirconia surface.

Based on the complete data set obtained, we propose three possible models as mechanisms of interaction of 10-MDP with zirconia, as schematically shown in Fig. 3. The left model (Fig. 3A) indicates that the 10-MDP monomer is adsorbed onto the zirconia surface via hydrogen bonding between the P=O (oxo group) and Zr-OH group. This was explained based on the peak at 0.3 ppm in the ^31^P MAS NMR spectrum (Fig. 2C)^27^. The second model indicates that the 10-MDP monomer may interact with zirconia via ionic bonding (Fig. 3B). The peak at −2.2 ppm in the ^31^P MAS NMR spectrum may represent this bonding state^32^. It is possible that one or two OH groups of 10-MDP deprotonated H^+^ to form P-O^−^ groups upon interaction of 10-MDP with zirconia^28^. Xie *et al*.^21^ simulated the chemical interaction between 10-MDP and zirconia using a numerical model; a double-coordinate and single-coordinate configuration was revealed. However, the in current study, ^31^P-NMR (Fig. 2C) revealed that P=O (oxo group) and one non-deprotonated P-OH of the PO_3_H_2_ group of 10-MDP might be engaged in hydrogen bonding either with zirconia or neighbouring phosphate groups.

It was reported that the pKa_1_ value for 10-MDP is 2.2 and that the pKa_2_ value is 7.0^33^. The 10-MDP primer probably causes the acid to have a neutral pH overall, even when 10-MDP interacts with zirconia; therefore, only one of the material’s P-OH groups can interact with either zirconia or the neighboring phosphate groups (Fig. 3B). The ^31^P MAS NMR data also supported this. There were two separate shifts at −7.7 and −2.6 ppm in ^31^P MAS NMR. Both two shifts were related to P-OH group, but different chemical states. The model on the right (Fig. 3C) indicates that in addition to ionic bonding between 10-MDP and zirconia, the adsorbed 10-MDP monomers have hydrogen-bonding interactions with zirconia via P=O (oxo group), causing the lower-frequency resonance shift in δ (^31^P) (−7.7 ppm). The combination of the ^1^H NMR and 2D ^1^H → ^31^P HETCOR NMR spectroscopy analysis confirmed the different chemical states of 10-MDP bonding to zirconia. The current study revealed not only ionic bonding between 10-MDP and zirconia, but also hydrogen bonding. Therefore, the hypothesis of the study that the chemical interaction of 10-MDP relies solely on the ionic interaction of 10-MDP^−^ with partially positive zirconium was rejected.

We conclude from the results of this study that one P-OH group of the 10-MDP molecule interacts with zirconia, while the other OH group interacts with P=O of another neighboring 10-MDP molecule. In this study, when a large amount of 10-MDP was used, interaction among different 10-MDP molecules through intermolecular hydrogen bonding was observed; thus, the 10-MDP concentration should be optimized in future investigations.

## Methods

### Sample preparation for shear bond strength (SBS) and SEM

Sintered zirconia round bars (3.4-mm diameter, 3-mm thickness) and sintered zirconia plates (10 × 10 × 3 mm) were prepared by Tosoh (Tokyo, Japan). The zirconia round bars were sandblasted (Hiblaster Ovaljet, Shofu, Kyoto, Japan) with Al_2_O_3_ particles (50 μm, Shofu) to ensure that the cement bonded strongly to the sandblasted zirconia surface and would not fail during the SBS test. The sintered zirconia plates were solely polished with 15-μm diamond particles to minimize surface retention (Fig. 1B) and should be regarded as the zirconia surface of interest. The latter surface was not sandblasted so that the effect of the differently concentrated 10-MDP based primers on the SBS could be assessed. They were then washed with distilled water and acetone using an ultrasonic bath. The plates were subsequently irradiated with a Xe2 excimer lamp (UER20-172B, Ushio Electric, Tokyo, Japan) at an ultraviolet (UV) wavelength (λ = 172 nm) in ambient air to clean the surface^34^. Each plate (n = 10) was dipped in 2 ml of 0.1 ppb to 1 wt% 10-MDP in acetone for 1 h using glass vials that were closed with lids to prevent acetone evaporation. As a control group, plates without 10-MDP treatment were also prepared. All plates were washed with acetone two more times to remove the excess (unbound) functional monomer. After this, they were dried using nitrogen. The sandblasted zirconia bars were luted onto zirconia plates (one bar per plate; n = 10) with Clearfil Esthetic Cement (Kuraray Noritake Dental, Tokyo, Japan) using finger pressure (corresponding to a pressure of about 2.2 MPa). The resin-based composite cement was cured for 1 min using a G-Light Prima II Plus lamp (2800 mW/cm^2^ light irradiance; GC). A cement film thickness of about 25 μm was reached, as was measured using Feg-SEM (JSM-6701F, Jeol, Tokyo, Japan). All specimens were stored in distilled water for 24 h at 37 °C.

### Shear bond strength (SBS) test

The specimens were mounted on a material-testing machine (Model 5565, Instron, Canton, USA), and shear stress was applied at a cross-head speed of 0.5 mm/min. After SBS testing, all failed specimens were analyzed using a light microscope (40×) (SMZ-10, Nikon, Tokyo, Japan) to assess the fracture pattern, which was used to classify the samples as having failed ‘cohesively’, ‘adhesively’, or ‘mixed’ (involving both cohesive and adhesive failure regions). For statistical analysis, one-way analysis of variance (ANOVA) followed by Tukey’s multiple comparison test was used with α = 0.05.

### Nuclear Magnetic Resonance (NMR) measurements

To analyze the chemical interaction of 10-MDP with zirconia using NMR, 2 g of zirconium (IV) oxide nanopowder stabilized with 3 mol% yttria (mean powder grain diameter: ≤100 nm, surface area: 10–25 m^2^/g; Sigma Aldrich, St. Louis, MO, USA) was mixed with a 50-ml solution of 25 wt% 10-MDP (Kuraray Noritake Dental) in acetone for 20 min via ultrasonic agitation, prior to being left without agitation for 40 min. The 10-MDP coated particles were separated from the mixed solution by centrifugation followed by a thorough wash with acetone in order to remove excess unbound functional monomer. For comparison, zirconia powder without any treatment was also prepared.

### NMR methodology

The local structure around H and P in 10-MDP coated zirconia particles was examined by solid-state MAS NMR. The NMR experiments were carried out using an Agilent DD2 500 MHz NMR spectrometer (Agilent Technologies Inc., Santa Clara, CA, USA). A zirconia rotor with a diameter of 3.2 mm was used for the ^31^P and ^1^H MAS-NMR measurements. The rotor spinning frequency was 15 kHz. Direct polarization ^31^P MAS-NMR spectra were taken at 202.3 MHz with pulse lengths of 2.8 μs (pulse angle of π/2) and recycle delays of 90 s. The signals of approximately 790 pulses were accumulated with NH_4_H_2_PO_4_ as an external reference (1.0 ppm vs. 0 ppm 85% H_3_PO_4_). ^1^H high-power decoupling was used during the ^31^P acquisition. ^1^H MAS-NMR spectra were taken at 499.8 MHz with a pulse length of 1.15 μs (pulse angle of π/4) and 120 s recycle delays; here, the signals from approximately four pulses were accumulated with adamantane (C_10_H_16_) as an external reference (1.91 ppm vs. 0 ppm TMS). For comparison, the ^1^H MAS NMR spectrum of zirconia powder without any treatment and the ^31^P NMR spectrum of the 10-MDP monomer in an acetone solution were also measured.

Two-dimensional (2D) ^1^H → ^31^P heteronuclear correlation (HETCOR) experiments were performed using cross-polarization with a contact time of 3 ms, a recycle delay of 5 s and 96 scans per *t*_1_ increments; 64 *t*_1_ slices were acquired, where the rotor spinning frequency was maintained at 15 kHz.

Study repeatability was guaranteed by having conducted each NMR analysis twice.

## Additional Information

**How to cite this article:** Nagaoka, N. *et al*. Chemical interaction mechanism of 10-MDP with zirconia. *Sci. Rep.* **7**, 45563; doi: 10.1038/srep45563 (2017).

**Publisher's note:** Springer Nature remains neutral with regard to jurisdictional claims in published maps and institutional affiliations.
